# Translational Regulations in Response to Endoplasmic Reticulum Stress in Cancers

**DOI:** 10.3390/cells9030540

**Published:** 2020-02-26

**Authors:** Manon Jaud, Céline Philippe, Doriana Di Bella, Weiwei Tang, Stéphane Pyronnet, Henrik Laurell, Laurent Mazzolini, Kevin Rouault-Pierre, Christian Touriol

**Affiliations:** 1 Inserm UMR1037, CRCT (Cancer Research Center of Toulouse), F-31037 Toulouse, France; manon.jaud@inserm.fr (M.J.); stephane.pyronnet@inserm.fr (S.P.); laurent.mazzolini@inserm.fr (L.M.); 2 Université Toulouse III Paul-Sabatier, F-31000 Toulouse, France; henrik.laurell@inserm.fr; 3 Barts Cancer Institute, Queen Mary University of London, London E1 4NS, UK; c.philippe@qmul.ac.uk (C.P.); doriana.dibella@qmul.ac.uk (D.D.B.); weiwei.tang@qmul.ac.uk (W.T.); k.rouault-pierre@qmul.ac.uk (K.R.-P.); 4 Inserm UMR1048, I2MC (Institut des Maladies Métaboliques et Cardiovasculaires), BP 84225, CEDEX 04, 31 432 Toulouse, France; 5 CNRS ERL5294, CRCT, F-31037 Toulouse, France

**Keywords:** translation initiation, ER stress, unfolded protein response (UPR), IRES, uORF

## Abstract

During carcinogenesis, almost all the biological processes are modified in one way or another. Among these biological processes affected, anomalies in protein synthesis are common in cancers. Indeed, cancer cells are subjected to a wide range of stresses, which include physical injuries, hypoxia, nutrient starvation, as well as mitotic, oxidative or genotoxic stresses. All of these stresses will cause the accumulation of unfolded proteins in the Endoplasmic Reticulum (ER), which is a major organelle that is involved in protein synthesis, preservation of cellular homeostasis, and adaptation to unfavourable environment. The accumulation of unfolded proteins in the endoplasmic reticulum causes stress triggering an unfolded protein response in order to promote cell survival or to induce apoptosis in case of chronic stress. Transcription and also translational reprogramming are tightly controlled during the unfolded protein response to ensure selective gene expression. The majority of stresses, including ER stress, induce firstly a decrease in global protein synthesis accompanied by the induction of alternative mechanisms for initiating the translation of mRNA, later followed by a translational recovery. After a presentation of ER stress and the UPR response, we will briefly present the different modes of translation initiation, then address the specific translational regulatory mechanisms acting during reticulum stress in cancers and highlight the importance of translational control by ER stress in tumours.

## 1. Introduction

Over the years, eukaryotic cells have evolved different mechanisms to deal with stressful environments. Under stress conditions, eukaryotic cells activate adaptive pathways to restore cellular homeostasis and to save energy. Given that cells consume a large amount of their available energy for the process of translation and for protein folding, it is not surprising that most stresses cause an inhibition in global protein synthesis. Indeed, under stress conditions, the maintenance of routine translation machinery would be deleterious. Hence the synthesis of “housekeeping” proteins is paused in stressed cells, whereas the translation of a pool of proteins necessary for the adaptive stress response is maintained, via alternative mechanisms of translational initiation. This level of regulation is particularly important in stress conditions, as it enables a rapid change in the protein synthesis level both quantitatively and qualitatively to obtain a response that is relevant to the type of stress being induced.

This is particularly true in cells with high growth rates and elevated metabolic requirements such as cancer cells, which are exposed to environmental stresses because of inadequate vascularisation causing hypoxia, acidosis and nutrient starvation. All of these stresses have been reported to cause the accumulation of unfolded or misfolded proteins in the lumen of the endoplasmic reticulum (ER) and induce ER stress because the folding capacity of the ER is limited. ER stress triggers activation of the unfolded protein response (UPR), an adaptive reaction mediated by three molecular sensors present on the membrane of endoplasmic reticulum: Activated Transcription Factor 6 (ATF6), Inositol-Requiring Enzyme 1 (IRE1) and PKR-like ER kinase (PERK). UPR activation in cells alters both transcriptional and translational programs to coordinate adaptive and/or apoptotic responses.

Indeed, UPR aims to restore cellular homeostasis and to promote cell survival by inhibiting protein synthesis, improving protein folding ability, increasing the degradation of unfolded proteins. However, when damages are irreversible after intense and prolonged activation, UPR induces cell death. At the moment, the molecular determinants of the transition from survival to death are still unknown.

Even if PERK kinase activation, which leads to phosphorylation of eukaryotic translation initiation factor-2α (eIF2α), is required for the global translation reprogramming during ER stress, involvement of IRE1 in translational regulation has also been established. 

It should be noted that the regulation of translation is extremely complex, with many interconnected mechanisms. Our aim here is not to go into the details of the various translation initiation modes, but to give the reader an overview of the translation rewiring upon stress. After a brief presentation of Endoplasmic Reticulum stress and UPR response, a particularly complex mechanism but also well documented in the literature, we will focus on the translational regulatory mechanisms acting during this stress in cancers and highlight the importance of translational control in stress conditions.

## 2. Endoplasmic Reticulum Stress Signalling in Cancer

The endoplasmic reticulum (ER), which accounts for more than 50% of the cell’s membranes in certain cells, is the site of synthesis and modification of secreted and membrane-related proteins (up to 50% of all proteins in certain cell types) [1,2]. It represents, therefore, an important hub where proteins undergo very strict quality control ensuring that only properly folded proteins progress down the secretory pathway. Thus, all situations leading to an alteration of the ER function, including the accumulation of excess unfolded proteins in the lumen of endoplasmic reticulum, lead to ER stress. Given the harmful impact of unfolded proteins, it is crucial that cells adapt to an imbalance between the ER’s folding capacity and unfolded proteins accumulation. The physiological response caused by the accumulation of misfolded or unfolded proteins is commonly called unfolded protein response (UPR) [2,3].

During cancer development and progression, cells are under a wide range of stresses, which include changes in oxygen levels (hypoxia), acidosis, nutrient starvation, disrupted calcium homeostasis, genotoxic or oxidative stresses. All of these stresses induce an accumulation of unfolded proteins within the reticulum, thus activating the UPR. In addition, cells with high proliferation rate, such as cancer cells, have to sustain a high rate of protein synthesis and massive protein flow through ER, leading to accumulation of misfolded protein in the ER, perturbation of ER homeostasis and finally to ER stress [4]. It has also been shown that genetic alterations found in cancers (translocations, mutations, aneuploidy, etc.) could be linked to the establishment of chronic ER stress [5,6,7]. ER stress and UPR are, therefore, the focal point of a large number of endogenous or exogenous cellular stresses. 

### 2.1. The Unfolded Protein Response

**Figure 1:**
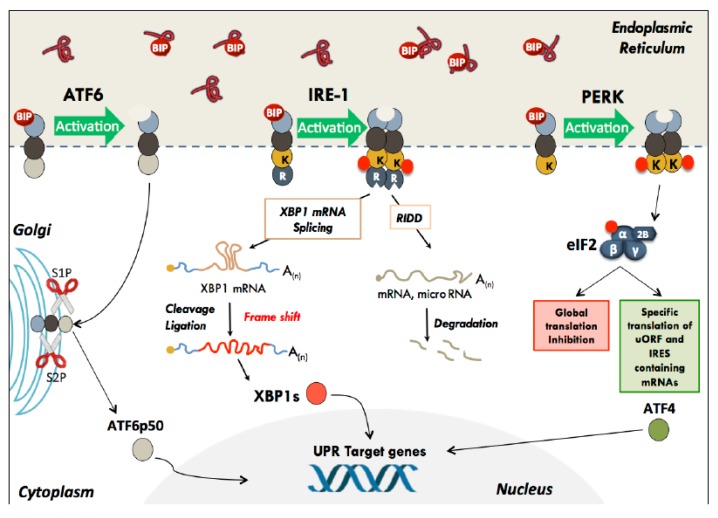
The different UPR effectors and their modes of action. In the basal state, the three UPR effector transmembrane proteins (PERK, ATF6, and IRE-1) are maintained inactive through their interaction with the protein chaperone BiP. The accumulation of poorly folded polypeptides in the ER lumen results in dissociation of BiP and activation of UPR. –I- PERK dimerises and phosphorylates the eIF2α subunit, leading to a global translation initiation inhibition. Specific mRNA subsets, containing cis-acting elements in their 5′UTR, such as uORF and IRES, escape translational inhibition triggered by eIF2 phosphorylation. –II- IRE-1 initiates an unconventional splicing of XBP-1 mRNA, as well as the degradation of some RNAs (this mechanism has been called RIDD for Regulated Ire1-Dependent Decay) –III- ATF6 traffics to the Golgi where proteolysis liberates its transcription factor amino-terminal domain, which is nuclearised and activates the expression of target genes.

In mammals, UPR is triggered by activation of three ER transmembrane sensors: PERK, ATF6, and IRE1 [3,8,9]. The luminal part of these proteins integrates the information coming from the ER lumen whereas their cytoplasmic part interacts with the effectors and mediates the signalling cascades (Figure 1). In absence of stress, the ER resident protein chaperone BiP interacts with the luminal domain of the three effectors and keep them in an inactive state. Upon accumulation of unfolded proteins in the ER lumen, BiP will act as a protein chaperone. Indeed, BiP has a relatively low but very broad affinity for hydrophobic regions of proteins, enabling it to recognise and bind a wide range of misfolded proteins exposing hydrophobic segments. As a consequence of its binding to the misfolded proteins, BiP is released from ATF6, IRE1, and PERK, leading to their activation [3,8,9]. 

Each of the three activated pathways will contribute, sometimes in a redundant way, to the stress response. Indeed, activation of these 3 sensors, namely IRE1, ATF6, and PERK, induce protective feedback mechanism essential to restore ER homeostasis through translation inhibition, to reduce protein synthesis, enhancing degradation of misfolded proteins and transcriptional regulation of specific stress target genes.

More generally, it seems that many chaperone proteins, residing in the endoplasmic reticulum, may be involved in the regulation of the activation/inactivation of UPR sensors. First of all, the involvement of different proteins of the PDI family (Protein Disulfite Isomerase) has been highlighted. PDIA5 has been described to interact with the ATF6 protein by modifying its conformation and consequently facilitating its export from ER [10]. Similarly, PDIA6 binds the disulfide bridges of the active IRE1 and PERK proteins and promotes their inactivation [11]. More recently, an original mechanism for activating IRE1 has been identified. The chaperone protein HSP47 directly binds the luminal domain of IRE1 and dislodges the BiP protein [12]. This mechanism promotes the activation of the IRE1 protein. Thus, HSP47 deficiency sensitises the cells to endoplasmic reticulum stress. Through screening of different IRE1 protein partners, this study revealed that other proteins such as mitochondrial ATPase Atp5h or phosphatase PP2a, are able to regulate IRE1 activity [12]. It has also been proposed that unfolded proteins may bind IRE1 thus promoting its oligomerisation and potentially modifying its activity [13].

The activation of the three UPR sensors, therefore, seems finely regulated and a better understanding of the mechanisms involved in these regulations could eventually allow each of these pathways to be specifically modulated. 

However, after activation of these three pathways, UPR fosters cell survival in response to stress in the following three ways: (i) blockade of protein translation to re-establish homeostasis; (ii) positive regulation of molecular chaperones to promote protein folding (iii) up-regulation of signalling pathways responsible for targeting ER misfolded proteins to degradation after ubiquitination. In addition, to promoting cellular survival, UPR can however also induce apoptosis under chronic or unresolved ER stress [14,15].

### 2.2. ATF6

ATF6 is a basic Leucine Zipper transcription factor (bZIP) with two isoforms, ATF6α and β. ATF6β is a distant homologue of ATF6α, and both are ubiquitously expressed. These two isoforms differ in their transactivation domain. As a result, a modulation of transcriptional activity is observed between the two proteins and is lower in the case of ATF6β [16]. The individual Knock-Out (KO) of each of these genes does not cause embryonic lethality [17]. In contrast, the double KO ATF6α/β^−/−^ triggers an embryonic lethality at E8.5, suggesting that some mutual compensations may be established between the two isoforms [17]. Unlike the other two sensors (PERK and IRE1), ATF6 is both a sensor and a direct effector of the UPR. Indeed, during stress, release of BiP unmasks a *Golgi* Localisation Signal (or GLS) in its C-terminal intra-luminal part [18]. ATF6 is then addressed to the Golgi and processed by two proteases (S1P and S2P) into an active ATF6p50 transcription factor [19]. Thus activated, ATF6p50 is nuclearised and participates in the transcription of stress response genes whose promoter contains UPRE (Unfolded Protein Response Element) or ERSE (ER Stress–Response Element) nucleotide motifs elements [19]. It activates specific transcriptional programs involved in (i) ER folding capacities enhancement by activating chaperone proteins [20,21], and (ii) increased protein turnover through the Endoplasmic Reticulum Associated Degradation system (ERAD) by upregulating genes such as EDEM (ER Degradation Enhancing alpha-Mannosidase like protein) or HERP (Homocysteine-responsive ER-resident ubiquitin-like domain member 1 Protein) [17]. ATF6 also activates expression of several transcription factors such as CHOP (C/EBP homologous protein) and XBP1(X-Box Binding Protein 1) [22,23]. 

ATF6 has been associated with cancer development, however its role in tumours has not been fully elucidated yet. In chronic myeloid leukemia, ATF6 drives cell survival upon imatinib treatment [10]. Some evidences also showed that ATF6 plays an important role on cell dormancy in rapamycin-treated tumours [24]. All together, these findings shed light on the potential role of ATF6 in chemoresistance.

### 2.3. IRE-1

IRE1 is the most conserved UPR sensor in eukaryotic cells, and is also the only one that has an embryonic lethal knockout phenotype at E12.5, resulting from a defective placental vascularisation [25]. The mammalian genome encodes two IRE1 isoforms, IRE1α and IRE1β. The first one is ubiquitously expressed while IRE1β expression is restricted to intestinal epithelial cells [26] and airway mucous cells [27].

IRE1 is a bifunctional protein, characterised by two cytoplasmic catalytic domains in its carboxy-terminal part: a serine/threonine kinase domain fused to an endoribonuclease domain (RNAse). During endoplasmic reticulum stress, protein dimerisation/oligomerisation triggers trans-autophosphorylation of the kinase domains, thereby inducing a conformational change leading to the allosteric activation of the RNase domain [14]. IRE1 activates several downstream intracellular signalling pathways through its RNAse activity and through its kinase activity. Indeed, it has been reported that the kinase domain is able to recruit the protein TRAF2 (TNF receptor-associated factor 2). The IRE1/TRAF2 complex then interacts with ASK1 (apoptosis signal-regulating kinase 1) to activate the JNK, c-Jun N-terminal kinase thus activating the pro-apoptotic ASK1/JNK (c-Jun N-terminal kinase pathway) [28,29].

The IRE1 endoribonuclease activity was first described for its role in cytoplasmic splicing of XBP1 (X-Box Binding Protein 1) mRNA. Once activated, IRE1 initiates the non-conventional XBP1 splicing by cleaving the mRNA at two sites in a conserved stem-loop structure folded sequence located in the open reading frame [30]. The excised sequence, whose length differs depending on the species, is composed of 26 nucleotides in humans. 

Then, the cleaved mRNA is processed by the tRNA ligase RTCB [31]. This unconventional splicing results in a frame-shift that allows the expression of an extended protein encompassing the transactivation domain of the transcription factor: XBP1s (s for spliced). The proteins XBP1s and XBP1u (u for unspliced), therefore, only differ by the presence or absence of the transactivation domain located in the C-terminal part, which also influences the stability of the proteins.

XBP1 splicing by IRE1 is a co-translational mechanism [32,33,34]. The nascent XBP1 protein possesses a highly hydrophobic domain (HR2), which enables the RNA/protein complex to be addressed to the endoplasmic reticulum membrane. More recently, it has been proposed that this relocalisation could happen indirectly through the recognition of the HR2 domain by the SRP (Signal Recognition Particle), which addresses the XBP1 mRNA to the IRE1/SEC61 complex [32]. A translational pause site would also facilitate this step [33].

Once activated, XBP1s induces the transcription of target genes for stress response by binding to UPRE or ERSE sequences in the promoter, but ChIP-seq studies have also shown that the role of XBP1s can extend to other cellular processes such as differentiation [35].

The endoribonuclease activity of IRE1 has been implicated in an additional process. For some other RNA targets, including both non-coding and coding RNA, the endonucleolytic cleavage by IRE1, sensitises these transcripts to the action of cytoplasmic exonucleases, triggering RNA decay. This mechanism, first described in Drosophila, is termed RIDD for Regulated IRE1-Dependent Decay [36]. Currently, only few direct targets have been characterised, however transcriptomic and bioinformatic studies seem to reveal a much broader spectrum of action [37,38]. Among the characterised RNA targets are 4 microRNAs (miR-17, 96, 125b, 34a) [39] and some mRNAs including PER1 [40], SPARC [41], BLOS1 [42], and DR5 [43]. Prediction of the RNA targets is relatively complex because no consensus sequence has been clearly defined even though an “XBP1-like” stem loop structure can be found at the cleavage site [44]. 

It remains to be determined how IRE1 selects between XBP1 splicing and RIDD. Given that both dimers and IRE1 oligomers have been detected during ER stress [45,46,47], it is likely that the RIDD mechanism can be influenced by IRE1’s oligomerisation status. Indeed, in addition to its intra-luminal dimerisation domain, IRE1 also contains a cytoplasmic oligomerisation domain leading to the formation of clusters that can be visualised as foci at the ER level during UPR [47]. While some studies showed that a high oligomerisation is correlated with activation of the RIDD [48], other experiments revealed an opposite effect [47]. Therefore, the precise impact of IRE1 oligomerisation status on XBP1 splicing and RIDD awaits further clarification.

IRE1 mutations can be found in cancers however the biological significance remains to be determined [15]. For instance, in glioblastoma, IRE1 has been widely investigated and shown to contribute to cancer progression by different mechanisms such as promotion of angiogenesis, tumour invasion and also inflammation [49]. Whereas, another study reported a negative regulation of invasion by IRE1 in a glioblastoma model [50].

Interestingly the main downstream effector of IRE1, XBP1 has also been found mutated in cancer [51]. Despite recent efforts to investigate the RIDD branch, XBP1 remains the most described target of IRE1 and thus the most studied in cancer, so far. In triple-negative breast cancers, upon hypoxia, XBP1 cooperates with HIF1a to promote tumour growth and foster relapse by activating pathways such as angiogenesis and glucose metabolism [52]. However, the pro-tumoural or anti-tumoural role of XBP1 in cancer is discussed and is probably context-dependent. In Multiple Myeloma, for instance, a high expression of XBP1s correlates with a lower response to thalidomide-based treatment [53] while a high XBP1s expression correlated with a better response to Bortezomib-based chemotherapy [54]. Moreover, two inactivating mutations of XBP1 have been characterised in multiple myeloma patients and are responsible for resistance to bortezomib treatment [55,56]. Therefore, further investigations are required to understand the role of the canonical and non-canonical IRE1′s pathways in cancers.

### 2.4. PERK

PERK is found in all metazoans and has the same domain organisation as IRE1; both proteins share a structurally- and functionally-related intra-luminal sensor domain [57] but a different cytoplasmic domain. The PERK monomer (encoded by the EIF2AK3 gene for eukaryotic translation initiation factor 2-alpha kinase 3) is located inside the ER membrane. The intra-luminal N-terminal part of PERK binds to BiP, which prevents its dimerisation, while its cytoplasmic C-terminal region contains a serine/threonine protein kinase domain. During the UPR, BiP is released from PERK, thereby allowing dimerisation and trans-autophosphorylation at Thr-982, which endows PERK with its catalytic activity [58,59]. Activated PERK, in turn, phosphorylates the subunit of eukaryotic initiation factor eIF2α at serine 51. The final consequence of PERK activation is the decrease in protein synthesis by rapid and potent inhibition of global translational initiation. 

Other PERK substrates have been identified: (i) Nrf2 (Nuclear factor (erythroid-derived 2)-like 2) transcription factor, which is a master regulator of redox homeostasis and whose stability is increased after PERK-dependent phosphorylation of the threonine 80 leading to increased Nrf2 nuclear import [60]. (ii) FOXO3, one of the Forkhead transcription factor family members which regulates a set of genes that contribute to cellular homeostasis and whose activity is increased after its PERK-dependent phosphorylation of serines 261, 298, 301, 303 and 311 [61]. (iii) Multiple DAG (diacyglycerol) species, which are important second messengers [62]. 

A key point of the present review is the PERK-eIF2α UPR signalling pathway, which is the most studied and, therefore, the best characterised. It’s however important to note that three additional eIF2α kinases have been discovered in mammalian cells which can be activated by different stresses: PKR, GCN2, and HRI. PKR is activated by long double-strand RNA and thus senses viral double-stranded RNA in infected cells [63,64,65]. GCN2 (general control nonderepressible 2) is activated by amino acid starvation through binding to uncharged tRNAs [66], or by UV irradiation [67,68]. Heme-regulated inhibitor (HRI) is known to get activated in various stresses, such as heme deficiency or heat shock in erythroid cells [69]. 

Much evidence showed an essential role of PERK in cancer. The PERK-ATF4 pathway induces autophagy in MYC-induced lymphoma and support the transformation process and tumour growth [70,71]. Furthermore, the PERK pathway has been reported to trigger a multidrug resistance phenotype in different tumour types through the PERK/Nrf2/MRP1 axis [72]. PERK is also closely linked to the anti-oxidative response. Whereby, by limiting oxidative DNA damages, PERK has been shown to enhance tumour growth [60]. The PERK-ATF4 axis has also been associated with metastasis through the transcription of matrix metalloproteinases [73]. Preclinical use of PERK inhibitors has shown great efficiency in pancreatic cancers [74]. However, the complexity of the downstream network activated by PERK suggests that the effect of PERK activation needs to be addressed in a context-dependent manner.

## 3. Translational Regulation: Dealing with Endoplasmic Reticulum Stress

Many studies have supported the idea that the UPR requires translational reprogramming, in which protein synthesis is globally repressed and is accompanied by the preferential synthesis of a specific subset of mRNAs whose protein products are required for responding to ER stress [45,75,76]. Indeed, while IRE1-XBP1 and ATF6 are well known to elicit a transcriptional response, the PERK pathway mainly induces an overall translational shutdown response by phosphorylating eIF2α, but it also enables increased translation of many stress-related genes including the transcription factor ATF4, which mediates a secondary transcriptional response. Although global translation inhibition allows cellular resources to be preserved, an efficient synthesis of some factors is necessary to cope with the consequences of stress. Mammalian mRNAs whose expression is known to escape translational inhibition triggered by eIF2α phosphorylation contain specific features in their 5′ untranslated region including uORF or IRES. After a brief reminder of the basics of mRNA translation initiation, we will develop some examples of RNA whose translation is upregulated during ER stress in more detail in the following paragraph. We will also discuss studies demonstrating the involvement of IRE1 RNase activity in translational regulations during reticulum stress.

### 3.1. Canonical Cap-Dependent Translation Initiation

**Figure 2:**
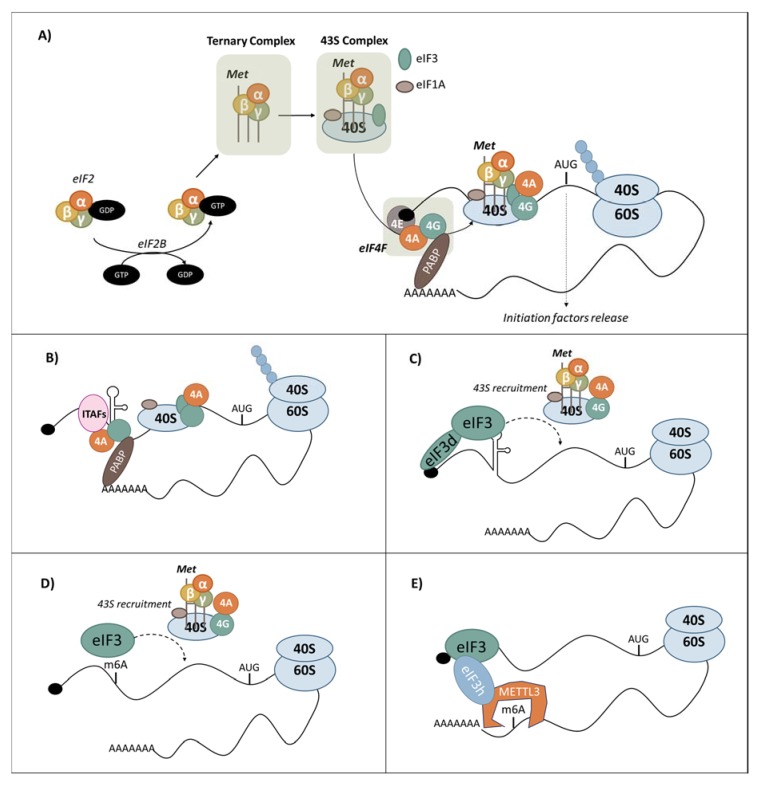
Currently known processes of translation initiation (**A**) Cap-dependent mechanism of translation. The eukaryotic initiation factor 2 (eIF2)-GDP is recycled in eIF2-GTP by the enzyme eIF2B. eIF2-GTP binds the methionine transfer RNA (Met-tRNAi) in order to form the ternary complex which integrates the 43S complex comprising the 40S ribosome subunit, eukaryotic initiation factors (eIF3, eIF1/1A) and the ternary complex. 43S is recruited to the mRNA through the m7G cap by interacting with the eIF4F complex (eIF4E, eIF4A, eIF4G) and 43S scans the 5′UTR until the first starting codon. The codon/anti-codon interaction triggers the release of initiation factors and the recruitment of the 60S, and then elongation can start. (**B**) Internal ribosome entry sites (IRES)-mediated translation initiation. The IRES directly recruits ribosomes, thereby bypassing the requirement of the mRNA 5′ cap structure. (**C**) The binding of the cap by the eIF3d subunit in presence of the stem-loop in the 5′ UTR can bypass the canonical eIF4E translation and initiate an eIF3d-directed cap-dependent mRNA translation. (**D**) A single 5′ UTR-located N(6)-methyladenosine m(6)A can promote cap-independent mRNA translation initiation, through direct interaction with eIF3 which is sufficient to recruit the 43S complex and initiate translation even in the absence of the cap-binding factor eIF4E. (**E**) METTL3 enhances translation of mRNA containing m(6)A in its 3′UTR through interaction with eIF3h.

Translation is a high demanding energy process, which needs to be rationalised upon stress conditions. The most well-known stress-relative pathways have been described to play critical roles in the regulation of the initiation step. In a nutshell, the general cap-dependent translation is turned off under stress conditions while the translation of some specific mRNAs is maintained or activated. Therefore, in order to understand the specific alternative routes of translation involved during stress, it is essential to understand the fine-tuning of the canonical cap-dependent translation initiation (Figure 2A). 

Ribosomes were identified as the link between mRNA and proteins in the 1950s, while the m7G-cap discovery came later with a first description in viruses in 1975 [77]. During those years, many efforts were made to identify this structure in eukaryotic mRNA and eventually led to the characterisation of the cap structure in HeLa and mouse myeloma cells [77]. The cap consists of a methylated guanine (m7G), which is engaged in an unconventional 5′ to 5′ triphosphate linkage to the mRNA. This structure plays an essential role in the mRNA stability and the regulation of translation initiation [77].

In order to interact with the cap, start scanning and initiate translation, the 40S small ribosome subunit (SSU) needs to be loaded. This priming is orchestrated by key actors of the translation, called eukaryotic initiation factors (eIFs): eIF3, eIF1/1A. In brief, eIF3 is binding the SSU in order to allow its recruitment at the cap. On the other hand, eIF1 and eIF1a are regulating the tRNA binding by stabilizing the preinitiation complex (PIC) in an open conformation. A third member of the eIFs family, eIF2 binds the SSU within a complex that also includes the methionine RNA transfer (Met-tRNAi), hence creating the ternary complex. The association of eIF2 and Met-tRNAi is allowed when eIF2 is loaded with a GTP. Therefore, the eIF2-GDP recycling into eIF2-GTP is a critical, rate-limiting, and highly regulated step, which is catalysed by the guanine exchange factor, eIF2B. At this stage, the SSU is comprised of eIF3, eIF1/1A and the ternary complex (eIF2-GTP-Met-tRNAi) thus forming the 43S pre-initiation complex [78,79,80,81]. 

The 43S PIC complex has the ability to bind the m7 GTP-cap through the heterotrimeric eIF4F complex composed of three non-identical subunits: the cap-binding protein eIF4E, the DEAD-box RNA helicase eIF4A, and the large “scaffold” protein eIF4G. This interaction recruits the PIC on the mRNA and the ATP-dependent scanning of the 5′UTR is initiated. When the first AUG enters the P-site of the SSU, the perfect codon/anti-codon matching triggers irreversible GTP hydrolysis in the ternary complex. Cooperative events are also required to fully complete the AUG recognition such as the release of eIF1 and 2. Then the binding of the 60S subunit to the 40S is catalysed by eIF5B-GTP and enables the first elongation step [78]. 

Although the 5′UTR plays a central role in the translation initiation process, it is important to mention that the 3′UTR is also involved in the regulation of translation initiation. Indeed, the poly(A)-binding protein (PABP) interacts with the cap through eIF4G and eIF4B, leading to the circularisation of the mRNA. A deregulation of this interaction can compromise translation [79]. Furthermore, circularisation of mRNA brings the 3′UTR binding regulators, such as micro-RNA or RNA binding proteins, next to the 5′UTR, giving them the ability to modulate the translation initiation step [81]. A key example is the mechanism of action of the Bicoid protein, which regulates the translation of mRNA during the development of Drosophila. Bicoid suppresses translation of caudal mRNA at the anterior of the embryo by binding the 3′UTR and an eIF4E-related protein, which compete with eIF4E in the binding of eIF4G [82].

### 3.2. Translational Control PERK-Mediated under ER Stress

Due to its central role during the recruitment of the initiator tRNA, the alpha subunit of eIF2, eIF2α, is one of the main targets for translation inhibition in the case of cellular stress and. as indicated previously represents the main PERK substrate. The phosphorylation of eIF2α on serine then induces overall translational impairment but mRNAs encoding stress response proteins must be able to escape global translation repression induced by PERK-mediated eIF2α phosphorylation, and many mechanisms have been developed to do so.

### 3.3. Selective mRNA Translation during eIF2 Phosphorylation:

#### 3.3.1. Regulation by uORF

##### Upstream Open Reading Frames: uORFs

uORFs represent one of the major regulatory motifs present in the 5′ UTR, which have been found to be involved in translational regulation under stressful conditions. In this context the repression imposed by uORFs on the initiation of translation of the main ORF is relieved, thus allowing the production of specific proteins in response to stress. The mode of action of each uORF appears dictated by its initiation codon context, secondary structure and coding capacities [83,84]. Moreover, the overall regulation of a given mRNA will depend on the specific combination and organisation of uORFs in its 5′ terminal region [85]. 

The precise mechanisms by which eIF2α phosphorylation regulates the relative translation initiation efficiencies at uORFs and at their downstream protein-coding sequences remain not entirely clarified and may be diverse depending on the nucleotide sequence and relative organisation of the different translation initiation regions [85]. For some genes which exhibit preferential translation during ER stress, such as ATF4 and C/EBPβ it has been proposed that during stress the lowered eIF2-GTP level resulting from eIF2α phosphorylation on Ser51 induces a reduction of the eIF2-GTP-met-tRNAi ternary complex intracellular concentration. This would delay the reacquisition of the ternary complex by the 40S ribosome after the translation reinitiation, which follows the completion of uORF translation and allow the scanning ribosome to skip downstream additional inhibitory uORFs sequences. This mode of regulation has been widely accepted by scientists in the field and has been proposed to take place in other stress-regulated genes as well [85]. However, kinetic and stoechiometric data validating this mechanism are still missing. Furthermore, the nucleotide sequence around the start codon of the uORF also influences the efficiency of translation of the downstream sequence and it has been shown that mRNAs which are preferentially translated upon stress frequently contain uORFs with suboptimal Kozak consensus sequences [86]. The phosphorylation of eIF2α on Ser51 may induce conformational changes, which impair initiation at AUG codons with suboptimal sequence context [87]. A decreased recognition of uORFs due to their unfavourable Kozak context has been proposed to be at least in part involved in the increase of expression of GADD34 [88] and CHOP [89,90] during ER stress.

It is currently assumed that the translation of uORFs is detrimental to the expression of the downstream coding sequences. These uORFs may exert their repressive effect by diverse, non-exclusive, mechanisms such as direct competition for translation initiation, translation elongation stall [89], and increased ribosome release for the translated mRNA [88]. In addition, uORFs could contribute to the inhibition of downstream CDS expression through mechanisms unrelated to mRNA translation initiation per se, such as increased mRNA decay [85,91]. However, a recent elegant research work demonstrated that most of the uORFs may actually remain translated during stress [92] and in addition that translational activation of the main coding sequence may involve at least in part specific translation initiation factors such as eIF2A [92]. Therefore, additional studies are still needed to fully elucidate the precise mechanisms of translational activation during ER stress.

A recent study illustrated well the relevance of investigating uORF in cancers. From different tumour samples, a screen based on a multiplex identifier-tagged deep sequencing, revealed 404 uORF and two loss-of-function mutations in uORF in EPHB1 and MAP2K6. By luciferase- assay, the authors confirmed that the observed mutations lead to an increase of translation of the downstream reporter. In parallel, whole exome sequencing allows one to identify another 53 deleting mutations in uORF suggesting that uORF-associated mutations can contribute to rewire the translation in cancers [93].

##### uORFs Translation upon ER Stress

Some of the proteins that were shown to be upregulated through an uORF-dependent mechanism are known to play a central role in determining cell fate following stress induction. The ER chaperone BiP/Grp78, a master regulator of ER stress signalling, contains 2 uORFs encoded from non-canonical initiation codons and the translation of these ORFs, which involves at least in part the translation factor eIF2A, was found to be necessary for sustained BiP expression during stress and, therefore, general modulation of the stress signalling response [92]. The activating transcription factor 4 (ATF4) regulates the expression of a variety of cytoprotective genes acting in particular on amino acid metabolism and oxidative stress damage and which participate in the recovery of the cell from the injuries, which induced the stress response. ATF4 is however also involved in the activation of the transcription of CHOP (C/EBP Homologous Protein)/GADD153/DDIT3 [22,94,95], a transcription factor which when expressed to high levels can activate the transcription of a set of genes promoting caspase activation and cell death. On the other hand, ATF4 and CHOP both up-regulate the expression of GADD34 [96,97], an activatory subunit of the protein phosphatase 1 complex, which is involved in the dephosphorylation of α and resumption of translation. Moreover, the combined action of the ATF4 and CHOP transcription factors at the promoters of a set of genes involved in protein synthesis has also been found to contribute to the re-induction of global translation [98]. Reactivation of protein translation following stress may have opposing effects on cell viability depending on the cellular context. Whereas resumed translation will participate in cell recovery after stress-associated injuries have been repaired, increased protein translation can also lead to cell death resulting from ATP depletion and oxidative stress [98]. Therefore, the precise and timely-controlled expression of factors such as ATF4, CHOP, and GADD34 regulating global translation as well as repair of cell damage or entry into apoptosis is essential to the cell in order to adequately cope with the stress response.

Interestingly, the mRNAs coding for these different proteins have all been found to contain functional uORFs that inhibit their translation under normal conditions. Upon stress induction however the inhibition imposed by the uORFs on ATF4, CHOP, and GADD34 is strongly abrogated and this mostly contributes to the re-expression of these different factors in the cell. Two uORFs are present in the 5′ region of ATF4. The most downstream uORF overlaps with the ATF4 coding sequence and inhibits its translation in normal conditions and low eIF2α phosphorylation [99,100]. A unique uORF encoding a specific peptide motif able to stall translation elongation and to inhibit CHOP translation in the absence of stress has also been identified in its mRNA [90]. The mRNA encoding GADD34 contains two uORFs. The most 5′ proximal sequence acts as a slight attenuator of GADD34 translation whereas the second uORF strongly inhibits downstream protein translation by likely promoting release of the ribosome from the mRNA after the uORF STOP codon has been reached [88,101].

The recognition and translation of the uORF may however in some instances be required for the up-regulation of proteins during stress. This is the case for the C/EBPβ transcription factor, which is expressed as three different isoforms produced from translation events occurring at different initiation codons. The shortest C/EBPβ isoform (LIP: liver-enriched inhibitory protein) has been reported to interact as an heterodimer with the CHOP transcription factor and to be essential to its nuclear relocalisation and the activation of its target genes, resulting in the induction of apoptosis [102]. The highly increased production of LIP during sustained endoplasmic reticulum stress involves the translation of an uORF allowing the ribosome to skip the initiation codon of the following main C/EBPβ isoform (LAP: liver-enriched activatory protein) and start translation from the LIP initiation codon [103].

Additional proteins involved in the metabolic recovery of cells and whose expression is induced during stress through an uORF-dependent manner have been identified. For example, the aminoacyl tRNA synthetase EPRS is involved in the charging of tRNAs with glutamine and proline residues, therefore restoring the available glutamyl- and prolyl-tRNA intracellular pools. Two inhibitory uORFs initiated at non-canonical codons (CUG and UUG) have been identified in the EPRS mRNA and shown to reduce EPRS translation under normal conditions [104]. This inhibition is relieved during stress, leading to the re-accumulation of EPRS in the cell. The amino acid transporter Cat-1, which mediates the uptake of the essential amino acids arginine and lysine, is induced during ER stress by different complementary mechanisms [105]. In particular, the translation of an uORF has been shown to play an essential role in the re-expression of Cat-1 by an original mechanism which involves the modification of the folding and reactivation of a cryptic IRES element in the 5′UTR of Cat-1 mRNA [106].

The currently available data strongly emphasise the essential role played by the upstream ORFs in the translational control of genes involved in the recovery from cell injuries and in the regulation of the stress response itself. Whatever the mechanisms operating, the uORF-dependent regulation of translation appears tightly controlled by the intracellular levels of phospho^51^-eiIF2α, which can be rapidly modulated during the stress response. It therefore represents a highly sensitive and responsive mean to regulate specific protein expression under stress conditions. Mutations affecting the activity of uORF and consequently the translation of the downstream ORF have been reported in different cancer models [83]. It is likely that uORFs mutations could as well affect the expression of stress-regulated factors and favour some cancer-associated processes but this has still not been reported to date.

#### 3.3.2. Cap-Independent Translation Regulation

##### IRES-Dependent Translation Initiation

In the early 80s, the cap-dependent mechanism was believed to be the only possible mechanism of translation initiation in eukaryotic cells. An alternative route that bypasses the initial cap-recognition, allowing ribosomal recruitment to internal locations in mRNA, termed internal ribosome entry sites (IRESs) was however revealed more than 30 years ago (Figure 2B) [107,108,109]. The IRESs were first discovered in the late 1980s in studies on poliovirus [108,110], whose characteristics are incompatible with cap-dependent translation initiation and encephalomyocarditis virus (EMCV) [109]. Indeed, poliovirus mRNA is naturally uncapped and the virus itself interferes with the cellular translation by proteolytic degradation of the cap-binding proteins, implicating that poliovirus mRNAs must be translated by cap-independent mechanisms. Many groups confirmed this notion for example by showing, through means of mutagenesis, that an internal sequence in the 5′UTR of poliovirus RNA was responsible for its cap-independent translation and this sequence could also confer cap-independent translation to heterologous mRNAs [108,109,110,111]. During the same years, a cellular mRNA, encoding glucose-regulated protein 78 (GRP78)/immunoglobulin heavy-chain binding protein (BiP), was found to be translated at an increased rate in poliovirus-infected cells, while cap-dependent translation was inhibited [107], showing for the first time that translation initiation by an internal ribosome-binding mechanism was used by eukaryotic mRNAs [112,113]. Since that time, many viral and cellular IRESs have been reported and recorded in the IRESite database [114]. More recently, the existence of IRES elements in cellular mRNAs was investigated using a high throughput strategy, which highlighted the existence of thousands of sequences, allowing cap-independent translation, and showed that 10% of mRNAs could potentially be translated by a cap-independent mechanism [115]. Interestingly, many IRES-containing mRNAs encode proteins that are involved in proliferation, differentiation, and apoptosis and can be translated when the overall cellular protein synthesis is inhibited upon different stress conditions, including ER stress, apoptosis, viral infection, nutrient starvation and hypoxia [116] generating ongoing interest in the field of protein translation and its regulation.

Despite a growing list of IRES-containing mRNAs, the mechanism of internal initiation is still poorly understood. Given that many IRESs have been identified in conditions of inhibited cap-dependent initiation, the cap-binding protein eIF4E and the scaffolding protein eIF4G do not seem to be required for IRES-mediated translation [107,117], although, it has been reported that, for example, MYCL IRES requires both eIF4E and eIF4G for its translation [117]. Curiously, homologs of eIF4G, such as eIF4G1, eIF4G2 [113,118] and DAP5/p97 [118,119], are shown to be associated with polysomes in poliovirus-infected cells and, at the same time, to be required for the IRES-mediated translation of selected mRNAs following cellular stress [119], implicating that eIF4G proteins are needed in both 5′ cap-independent and 5′ cap-dependent translational initiation mechanisms. Finally, the activity of the RNA helicase eIF4A seems to be essential for the translation of MYC, MYCN [120] and BIP IRESs [121]. The role of eIF2 has also been investigated for cellular internal initiation. Importantly, IRES-mediated mRNAs translation can operate upon global protein synthesis attenuation induced by eIF2 phosphorylation [116,122,123,124,125,126,127]. In addition to the involvement of canonical initiation factors, efficient IRES-dependent translation requires auxiliary RNA-binding proteins, known as IRES trans-acting factors (ITAFs; Figure 2B) [128]. The mechanism of ITAF function is not fully understood, but it is generally believed that many ITAFs are required for the stabilisation of IRES conformation. Importantly, the subcellular localisation of ITAFs have been shown to be crucial for their function [128] (described below). Examples of ITAFs include La autoantigen [129] and several heterogeneous nuclear riboproteins (hnRNP) such as hnRNPC1/C2 [130] and hnRNPA1 [123,131]. 

One of the most relevant examples highlighting IRES importance in cancers is the MYC IRES in multiple myeloma. Indeed, a point mutation in this specific IRES sequence was identified in 42% of patient bone marrow samples [132]. This mutation enhances the translation of the proto-oncogene MYC suggesting that IRES deregulation could be responsible for overexpression of oncogenes. In silico analysis fail to reveal IRES that need to be functionally tested in-vitro, which render their identification laborious hence the list of IRES is still limited. However, IRESs have already been described in many other mRNAs encoding proteins involved in tumourigenesis and cell survival (Apaf-1, cJUN, AML1/Runx1, EGFR/HER1, BCL2, BCL-XL, XIAP, MYC, MYCN, VEGF-A, P27, P53), suggesting that IRES-mediated translation play a crucial role in tumour progression and survival [133]. Direct evidence that supports this hypothesis comes from many studies. For example, 3D spheroids culture of ovarian cancer cells treated with a PI3K/mTOR inhibitor reveals resistant cells expressing BCL2, which is indeed translated by a cap-independent mechanism in these conditions [134]. IRESs mediated translation also promotes inflammatory breast cancer tumour cell survival and formation of tumour emboli by activating p120 catenin mRNAs expression [135]. Another example concerns the P53 protein, which is also translated by a cap-independent mechanism due to the presence of two IRES residing within the 5′UTR and the coding sequence [136,137]. These IRES, activated in response to DNA damage, binds several ITAFs including DAP5 (or p97 or NAT1) which is a member of the eIF4G proteins, the translational control protein 80 (TCP80), Ribosomal protein L26 (RPL26) and nucleolin [138,139,140]. If these proteins are either over or underexpressed in the cells, or if mutations affect p53 mRNA IRES structure, p53 protein level can fluctuate [136,141]. Thus, some wild type p53-expressing cancer cells may not express p53 due to an IRES-dependent defective translation demonstrating the key role of IRES-mediated translation initiation in cancer development.

##### IRES Regulation in Response to Stress

Even if cellular IRESs have been described in a limited, but growing, number of mRNAs, many genes involved in stress response, such as HSPA5, ATF4, HIF1 α, NRF2, FGF-2, VEGF-A, VEGF-C, STAUFEN or DLL4 are thought to contain IRESs [123,142,143,144,145,146,147,148,149,150,151].

Several master regulators of the UPR can be translated by a cap-independent mechanism. Remarkably, the BiP transcript was the first cellular mRNA reported to contain an IRES [112]. Moreover, ATF4 translation is regulated by either uORFs or an IRES. Indeed, an alternatively spliced variant of ATF4, expressed in leukocytes and induced by UPR, is translated by a cap-independent mechanism, which is activated by PERK-mediated eIF2α phosphorylation [152]. The presence of these elements therefore allows these mRNAs to be efficiently translated in stress conditions. 

PERK activation also results in an IRES-dependent activation of TP53 isoform translation [153]. It has been demonstrated that two TP53 isoforms, TP53 and TP53/47, are translated by two different IRESs located on the same mRNA [137,154]. In stressful situations, an increase of the TP53/47 isoform-dependent IRES translation results in a cell cycle arrest in the G2 phase, while the full length TP53 induces a G1 phase arrest [153,154].

Cellular IRESs are often found in long and GC-rich structured 5′UTRs, and are relatively ineffective in directing translation under physiological conditions. However, the precise molecular mechanism of cellular IRES-directed translation in stress conditions when eIF2α phosphorylation is not completely understood.

Stabilisation of secondary structures, for example through interaction with proteins, could slow down the scanning of the 43S pre-initiation complex and thus promote translation re-initiation efficiency [155]. According to the “land and scan” initiation model used by the already characterised cellular IRESs, the 40S subunit lands at the IRES before scanning from 5′ to 3′ to the initiation codon [156,157,158]. Since IRESs are highly structured elements, one hypothesis is that these elements could represent a barrier that would slow down ribosome progression, thus explaining the increase in IRES-dependent translation under eIF2α phosphorylation conditions. In the same way, these structures, potentially bound by proteins, may also be pause sites allowing the ribosome to recruit active initiating factors, thus stimulating translation when eIF2α is phosphorylated.

As previously mentioned, most IRESs, require ITAFs for their regulation [159]. The expression and the activity of these ITAFs can be modulated by UPR. For example, under ER stress, caspase-12 cleavage of the ITAF eIF4G2 (DAP5/p97) produces a fragment known as p86 [160] enhancing the IRES-mediated translation of HIAP2 (human apoptosis protein 2 inhibitor) Apaf-1 and XIAP [161,162] leading to the reduction of apoptosis and allowing the UPR to cope with stress [163].

Subcellular relocalisation of ITAFs plays also a crucial role in the modulation of IRES-dependent translation efficiency [128]. This is the case of hnRNPA1, which is relocalised from the nucleus to the cytoplasm during ER stress [131]. Moreover, hnRNPA1 cytoplasmic accumulation requires eIF2α phosphorylation [164], and was shown to modulate IRES-dependent translation of SREBP-1a, c-MYC or DLL4 in response to endoplasmic reticulum stress [123,131,165].

Such relocation has also been documented for other ITAFs, including PTB and PCBP1 (poly r(C) 1 binding protein or hnRNPE), which work jointly to activate BAG1 IRES (Bcl-2 Associated with Athanogen 1) following chemotoxic stress [166]. Nucleolin is also translocated from the nucleolus to the cytoplasm and activates the VEGF-D IRES-dependent translation in response to heat shock [167]. Interestingly both chemotoxic stress and heat shock are known to activate the UPR.

##### The Angiogenesis Paradigm

Angiogenesis is critical for many physiological processes, such as embryonic development and wound healing, but also in pathological states including the development of solid tumours.

As previously indicated, more than 100 mammalian mRNAs harbour IRESs in their 5′UTRs. Interestingly, these mRNA include many mRNA encoding proteins strongly involved in the angiogenic process like VEGF-A, VEGF-C, VEGF-D, FGF-2, HIF1α, DLL4 or TSP1 [123,142,148,167,168,169,170,171]. Angiogenesis depends on the highly coordinated action of a variety of angiogenic regulators, the most prominent and best characterised being Vascular Endothelial Growth Factor A (VEGF-A), Fibroblast Growth Factor 2 (FGF-2) and DLL4. Indeed, DLL4 is with VEGFA one of the few examples of haplo-insufficiency, resulting in obvious vascular abnormalities and in embryonic lethality [172,173,174].

It was already demonstrated that VEGF-A, FGF-2 and DLL4 IRESs are activated upon stress conditions including hypoxia or ER stress and that these mRNAs remain efficiently translated under ER stress conditions despite phosphorylation of the major PERK substrate, eIF2α [123,124,142,168].

These results are consistent with the fact that tumours derived from K-Ras-transformed Perk^−/−^ MEFs (mouse embryonic fibroblasts) display less angiogenesis and grow less rapidly than tumours with an intact UPR signalling [175], demonstrating the role of PERK activation in the angiogenic process.

**Figure 3:**
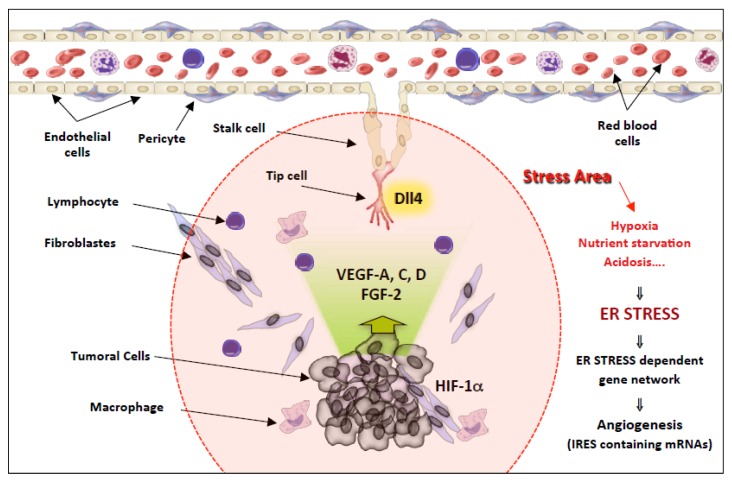
Schematic model of the network of gene expression co-regulation by IRES elements in stress conditions during tumoural progression. During tumour progression, the stress zone encompasses the growing tumour, but also its microenvironment. Both the tumour and the neo vessels, more particularly the Tip cells located at their extremity, which guide the neo vessels towards the tumour, are located in this unfavourable microenvironment. Hypoxia, nutrient starvation, and acidosis will irremediably induce the accumulation of unfolded protein in the reticulum of cells located in this area, leading to endoplasmic reticulum stress and UPR activation. Thus, in addition to transcriptional regulations, the activation of the PERK pathway will induce the co-regulation an UPR-dependent gene network containing IRES elements, revealing a translational regulon in which the synthesis of a cohort of angiogenic master regulator genes including VEGF-A,C,D, FGF-2, DLL4, and HIF1 is activated in response to ER stress. The fine-tuning of gene expression allows for efficient angiogenesis, which is a highly regulated process.

The presence of IRESs in many mRNAs encoding proteins tightly involved in the angiogenic process enables a selective co-regulation of these mRNAs expression under stress conditions (Figure 3). The tumour microenvironment is composed of a set of tumour and stromal cells and extracellular matrix. During tumour progression, impaired vascularisation causes several stresses including hypoxia, glucose or amino acid starvation or acidosis. These unfavorable conditions are known to induce ER stress, phosphorylation of eIF2α and thus activation of a gene network dependent on this phosphorylation in the stress area surrounding the tumour. Consequently, mRNAs encoding VEGF-A, -C, -D, FGF-2, HIF1A or DLL4 that are expressed by tumour cells or microenvironment (such as DLL4 expressed by TIP cells which are furthest away from the circulating blood are still efficiently translated while cap-dependent initiation is compromised (Figure 3). IRES therefore function as cis-acting regulons during ER stress.

These results demonstrate that for cancer to progress under stressful conditions, it must use alternative translation mechanisms, such as IRES-dependent translation, to promote angiogenesis and thus survival and growth.

### 3.4. mRNA Translational Control by IRE1 under ER Stress

Even if it is much more anecdotal, several studies have proposed that IRE1 also participates in translation repression during ER stress. 

First of all, IRE1 selectively suppresses secretory protein translation by targeting mRNAs through RIDD to alleviate the load on the protein folding machinery. As indicated previously, a few dozens of mRNAs are known to be targeted by IRE1. Even if a consensus cleavage site embedded in a stem loop structure has been identified in vitro [37,176,177], it remains particularly difficult to predict the direct RIDD targets. The question arises of how these RIDD mRNA targets are addressed to the reticulum membrane. Indeed, most but not all these mRNAs encode proteins with signal peptide/transmembrane domains [36]. It was demonstrated that the removal of the signal peptide from a known RIDD target impedes its degradation and, inversely, introduction of a signal peptide to the GFP mRNA is sufficient to favour its degradation by IRE1 [178]. However, the question of the targeting of messenger RNAs that do not code for secreted or membrane proteins remains open. Alternative mechanisms could, for example, involve specific RNA-binding proteins. It is, however, important to mention that mRNAs encoding cytoplasmic proteins are also found at the membrane of the ER [179,180]. In addition, ribosome-profiling experiments combined with subcellular fractionation demonstrated that ER-associated mRNAs encoding both cytosolic proteins and those encoding secreted/membrane proteins display similar ribosome loading densities [181]. These data, therefore, suggest that ER-associated ribosomes would play a major role in the translation of both mRNAs encoding cytoplasmic or secreted proteins. On the other hand, the co-activation of PERK and IRE1 pathways appears to be essential to the RIDD mechanism. Indeed, it has been shown that PERK depletion decreases the degradation of some mRNA targets while artificial translation blockage restores the RIDD. To explain these results, a possible hypothesis is that translation by ribosomes disturbs the stem loop structure recognised and cleaved by IRE1. Thus, the correct recognition of the target RNA would require a translation blockade by PERK or eventually a translational pause [182].

In glioma cells, IRE1 has been described to target the mRNA encoding the extracellular matrix protein SPARC. Downregulation of SPARC by IRE1 leads to a modulation of stress fibre formation and enhances migration properties of glioma cells [41]. Another substrate of the RIDD is PER1 mRNA, a circadian clock gene that controls the expression of CXCL3, an important chemokine involved in cancer development [40]. IRE1 has also been reported to protect cells from apoptosis, notably, through the decay of the death receptor 5 (DR5) mRNA. These few examples among the increasing list of RIDD targets highlight the pivotal role of this IRE1 downstream pathway in cancer [43].

IRE1 can also modulate the translation by another mechanism. Indeed, it was demonstrated that overexpression of IRE1β induces 28S rRNA cleavage [183] more efficiently than IRE1α [184]. In this inducible hIRE1β expression model, total protein synthesis was repressed by 30% 1 day after hIRE1β induction. Thus, the cleavage of 28S RNA could reduce the number of functional ribosomes. As it was demonstrated that reduced ribosome levels impaired the translation of transcripts that are normally efficiently translated and have short and unstructured 5′UTRs in comparison to other transcripts [185], this mechanism could enable the specific modulation of expression of certain messenger RNAs. 

Finally, a subset of RNAs including ER-targeted mRNAs, SRP RNA, ribosomal and transfer RNAs were demonstrated to physically associate with IRE1 in living cells [186]. Moreover, IRE1 interacts with the translocon, the translocon-associated TRAP component, SRP proteins and ribosomal proteins [32]. IRE1 also strongly binds 80S ribosomes in vitro [186]. These results show that IRE1 is closely coupled to the translation machinery, but the precise functions of these different interactions are not yet clearly identified.

Although post-transcriptional gene regulation mediated by the IRE1 proteins has received a lot of attention in recent years, our understanding of the role of IRE1 in translational regulation is still in its early days. 

### 3.5. mRNA Structures and Modifications Regulate Translation:

#### 3.5.1. eIF3 Recruitment by RNA Structures

The multisubunit initiation factor eIF3 (13 subunits eIF3a-m) plays a central role in the cap-dependent translation initiation through its interaction with eIF4G, which allows the recruitment of the 43S pre-initiation complex [187]. However, eIF3 can also interact with mRNA stem loop structures in the 5′UTR and directly regulate both cap-dependent and independent translation. eIF3 plays an essential role in translation of specific subsets of mRNA [188]. Indeed, Lee et al., reported that eIF3 uses different modes of mRNA stem loop binding to exert positive and negative translation regulation of key proliferative transcription factors such as cJUN and BTG1 [188]. Using PAR-CLiP (photoactivatable ribonucleoside-enhanced crosslinking and immunoprecipitation) technology, they showed that eIF3 interacts with 3% of the expressed transcripts through direct interactions of mRNA with the eIF3 subunits a, b, d and g. The binding of the cap by the eIF3d subunit in presence of the stem loop in the 5′UTR allows to bypass the canonical eIF4E translation and initiate an eIF3d-directed cap-dependent mRNA translation (Figure 2C) [189].

Different viruses seem to exploit the capacity of eIF3 to initiate translation. The hepatitis C virus harbours an IRES whose direct interaction with eIF3 is critical to induce efficient translation initiation [190]. The positive strand RNA virus, Barley Yellow Dwarf Virus (BYDV, Genus Luteovirus) employs a cap-independent mechanisms where eIF3 bridges the mRNA 5′ and 3′UTR to initiate and regulate its translation [191]. 

There is still limited evidence of a role of this eiF3-dependent translation regulation mechanism under ER conditions. For example, it was demonstrated that mutations in eIF3k and eIF3l genes enhanced resistance to ER stress in *Caenorhabditis elegans* [192]. Moreover, UV crosslink experiments reveal that activation of ER stress by thapsigargine treatment results in a marked increase of several eIF3 subunits binding to polyadenylated mRNAs [193]. This work also demonstrates that eIF3 subunits favour the recruitment of selected mRNAs to 40S ribosomes during chronic ER and that this chronic stress renders eIF3 as the key mediator of mRNA recruitment to the PIC [193]. Taken together, even though the molecular mechanisms are still unknown, these data suggest a critical functional role for these eIF3 subunits in the regulation of cellular responses to ER stress.

#### 3.5.2. m6A-Dependent Translation Initiation

In addition to secondary mRNA structures, specific RNA modifications may have a strong impact on alternative translation mechanisms. For instance, a single N(6)-methyladenosine (m(6)A) residue in the 5′UTR promotes cap-independent mRNA translation initiation, through direct interaction with eIF3 which is sufficient to recruit the 43S complex and initiate translation even in the absence of the cap-binding factor eIF4E [194] (Figure 2D). 

N(6)-methyladenosine modification is the most abundant post-transcriptional mRNA modification [195,196], it exhibits tissue-specific regulation with an enrichment of m(6)A sites near stop codons and in 3′UTRs [197,198]. The methylation and demethylation of mRNA adenosine is dictated by “writers” and “readers” (reviewed by [199]). The intracellular fate of methylated mRNA is under the control of “readers” which according to their abundance, localisation (nucleus vs. cytoplasm), or the presence of specific RNA binding proteins (RBPs) will determine mRNA decay, stability or translation [187,199]. Thus, three readers—YTHDF1, YTHDF3, and YTHDC2—are heavily involved in translation of m(6)A mRNA [200,201,202]. YTHDF1 selectively recognises m(6)A 3′ UTR modified mRNA, promotes ribosome loading and interacts with different subunits of eIF3 complex to facilitate translation initiation [202]. YTHDF3 promotes protein synthesis in synergy with YTHDF1 and affects methylated mRNA decay mediated through YTHDF2 (“reader” involved in mRNA decay). However, it is still unclear if the processes require direct interaction between the different YTH proteins or the cooperation of co-factors [201]. While decreasing the abundance of m(6)A 3′UTR modified mRNAs, YTHDC2 also enhances their translation efficiency. The authors suggested that YTHDC2 may increase the translation of transcripts, and then destabilise the transcripts after translation has been completed to prevent further differentiation of cells [200].

Interestingly while METTL3 is a m(6)A “writer” in the nucleus, it functions as a potential “reader” when localised in the cytoplasm where it enhances translation of m(6)A mRNA through interaction with eIF3h (Figure 2E). METTL3 has also been proposed to promote oncogene translation through a mRNA looping mechanism [203,204]. In addition, promoter-bound METTL3 can induce m(6)A modification within the coding region of the associated mRNA transcripts, and enhance its translation by relieving ribosome stalling [205].

Although N6-methyladenosine marks on mRNA are preferentially located in the 3′UTR [197], diverse cellular stresses can induce a wide redistribution of m(6)A transcriptome marks, resulting in increased numbers of mRNAs with 5′UTR m(6)A [194]. For instance, heat shock stress induces preferential m(6)A deposition at the 5′UTR of newly transcripts. In the nucleus, under such stress, YTHDF2 preserves 5′UTR methylation of stress-induced transcripts by limiting the m(6)A “eraser” FTO activity. This mechanism allows the cap-independent translation of stress mRNA such as Hsp70 [198]. Moreover, it was already reported that m(6)A could recruit eIF3 to induce 48S initiation complex formation independent of eIF4E cap binding [194] especially under heat shock conditions when cap-dependent initiation is blocked, and also that m(6)A-based regulation contributed to the translational control of *ATF4* during amino acid starvation [206]. A correlation between increased local m(6)A modification and usage of non-canonical start codons during amino acid starvation was also described, which increase further the complexity of translation regulation mechanisms in stress conditions [206]. Even though the reversible m6A RNA methylation process has been now widely described, the cellular functions of this modification remain largely unclear, and further studies will be required to clarify the role of this modification in translational regulation, specifically during ER stress.

## 4. Conclusions

Protein translation regulation is an essential mechanism to maintain the cell’s integrity and enable it to cope with stresses. Protein synthesis is not a single process, but a combination of several different mechanisms that finely regulate the expression of specific mRNAs in response to stress in order to quickly adapt the cellular proteome. 

Upon ER stress and eIF2α phosphorylation by PERK, cancer cells use alternative translation mechanisms that are mediated by cis-acting sequences, such as uORF and IRES, to drive the expression of specific mRNA subsets involved in the stress response. Even if translational regulation mediated by uORF and IRES during stress response is well documented, these alternative mechanisms of translation are not yet fully understood.

Thus, even if the knowledge of translational regulations has grown considerably during the past few years, thanks in part to recent technological advances in profiling genome-wide translation, strong efforts must be made in order to better understand the molecular mechanisms underlying translational control in cancer during the stress response. In addition, we believe that a better understanding of the mechanisms allowing the selective translation of specific mRNAs in stress conditions could also lead to the identification of new targets and holds great promise for novel therapeutics in oncology.
